# A Close View of the Production of Bioactive Fungal Metabolites Mediated by Chromatin Modifiers

**DOI:** 10.3390/molecules29153536

**Published:** 2024-07-27

**Authors:** Jacqueline Aparecida Takahashi, Laura Lima de Queiroz, Diogo Montes Vidal

**Affiliations:** Departamento de Química, ICEx, Universidade Federal de Minas Gerais, Belo Horizonte 31270-901, MG, Brazil; lauralimadq@ufmg.br (L.L.d.Q.); vidal@qui.ufmg.br (D.M.V.)

**Keywords:** natural products, epigenetic, OSMAC (one strain, many compounds), fermentation protocols

## Abstract

Secondary metabolites produced by fungi are well known for their biological properties, which play important roles in medicine. These metabolites aid in managing infections and treating chronic illnesses, thereby contributing substantially to human health improvement. Despite this extensive knowledge, the vast biodiversity and biosynthetic potential of fungi is still largely unexplored, highlighting the need for further research in natural products. In this review, several secondary metabolites of fungal origin are described, emphasizing novel structures and skeletons. The detection and characterization of these metabolites have been significantly facilitated by advancements in analytical systems, particularly modern hyphenated liquid chromatography/mass spectrometry. These improvements have primarily enhanced sensitivity, resolution, and analysis flow velocity. Since the in vitro production of novel metabolites is often lower than the re-isolation of known metabolites, understanding chromatin-based alterations in fungal gene expression can elucidate potential pathways for discovering new metabolites. Several protocols for inducing metabolite production from different strains are discussed, demonstrating the need for uniformity in experimental procedures to achieve consistent biosynthetic activation.

## 1. Introduction

Antitumor, immunosuppressant, antibiotic, and antilipidemic activities are among the many biological roles attributed to secondary metabolites of fungal origin [1,2,3,4]. These metabolites have provided invaluable benefits to humanity, such as overcoming once-fatal infections, increasing survival rates in transplant recipients, and controlling the severity of some non-communicable chronic diseases. However, these achievements seem to represent just the tip of the iceberg, given the estimated 1.5 million fungal species described by Hawksworth in 1991 [5], subsequently revised to 3.8 million [6], underscoring the potential to be explored [7,8]. In a recent review, Niskanen et al. (2023) proposed a new estimate of 2–3 million global fungal species, with at least 92.5% remaining unknown. These authors suggest that the process of describing new fungal species could take centuries [9]. Meanwhile, research on bioactive fungal metabolites is progressively increasing fueled by studying unconventional habitats for new species, such as frozen biomes [10], ocean sediments [11], oceanic trenches [1], alpine ecosystems [12], marine organisms [13,14], endolichenic fungi [3], and algicolous species [15], and even unconventional sources like bee ecosystems [16] and spider eggs sacs [17], among others. Additionally, fungi exhibit limitless biosynthetic creativity, producing new classes of metabolites and uncommon skeletons [18]. Cytochalasans [19], indole alkaloids [20], meroterpenoids [21], and polyketides [22,23] represent only a few examples of this diversity. This favorable scenario has been facilitated by advancements in techniques of cultivating, isolating, and bioprospecting bioactive secondary metabolite-producing species. These efforts aim to use their potential in the pharmaceutical and cosmetic industries [24,25,26] as well as in industrialized foods as additives or functional ingredients [27,28]. This review explores various aspects of natural product production by filamentous fungi, focusing on structural diversity and expression of silenced biosynthetic routes through fermentation process modifications. The importance of standardizing some fermentative parameters is also discussed, especially those related to cultivation time and concentration of epigenetic modulators used in the experiments.

## 2. The Production of Secondary Metabolites by Fungi

**Figure 1:**
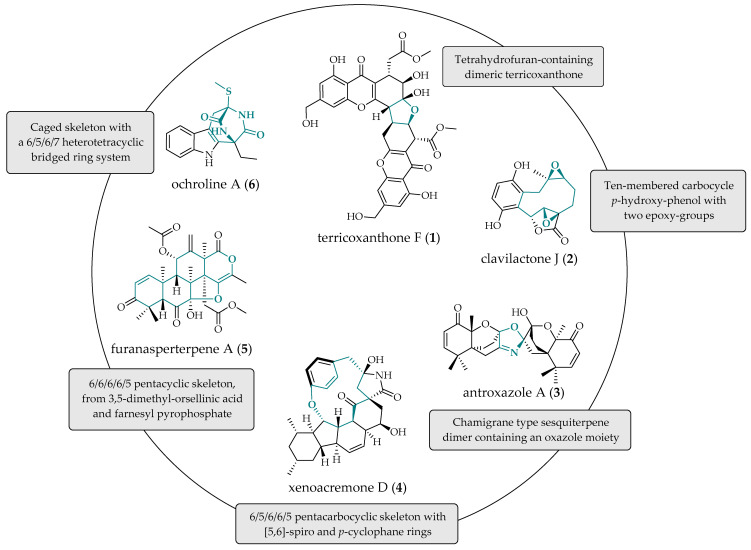
Structures of terricoxanthone F, clavilactone J, antroxazole A, xenoacremone D, furanasperterpene A, and ochroline A.

Secondary metabolites are small molecules produced by fungi, bacteria, plants, algae, and other organisms. Their biosynthesis can be governed by genes located in clusters [29,30] or by other complex pathways [22] and usually produces extracellular signaling molecules that support fungal survival in competitive microbial communities. While some secondary metabolites are ubiquitous and structurally simple, most fungal metabolites exhibit complex chemical structures, novel skeletons, unusual stereochemistry, and a wide range of pharmacological activities. The biosynthesis of structurally complex fungal secondary metabolites, including cyclic non-ribosomal hexapeptides like echinocandins [31], structurally diverse polyketides [32], azaphilones [33], including toxins [34], is well established. Figure 1 shows the chemical structures of terricoxanthone F (**1**), clavilactone J (**2**), antroxazole A (**3**), xenoacremone D (**4**), furanasperterpene A (**5**), and ochroline A (**6**), which are representative molecules of fungal origin with creative skeletons. The production of metabolites with high structural complexity, as demonstrated in these examples, is an important route for the development of new drugs for various applications, as illustrated below.

The dimeric xanthone terricoxanthone F (**1**) was isolated from *Neurospora terricola* HDF-Br-2, a fungal species associated with the plant *Pseudotsuga gaussenii*, as part of a program aimed at identifying bioactive metabolites from fungi associated with endangered Chinese coniferous plants. This new metabolite presented activity against *Candida albicans* (64 μg/mL), *Staphylococcus aureus* (128 μg/mL), and a mixed culture of *C. albicans* and *S. aureus* (128 μg/mL) during biofilm formation, as well as disrupting preformed *C. albicans* biofilms (128 μg/mL) [35]. Clavilactone J (**2**), a highly oxidized meroterpenoid isolated from the basidiomycete *Clitocybe clavipes*, inhibits the human gastric cell line HGC-27 (IC50 56.6 ± 1.34 µM) [36]. Antroxazole A (**3**), an oxazole-containing chamigrane sesquiterpene isolated from *Antrodiella albocinnamomea*, exhibits immunosuppressive activity by inhibiting lipopolysaccharide-induced (LPS-induced) proliferation of B lymphocyte cells (IC50 16.3 µM) [37]. Xenoacremone D (**4**) is a novel polyketide nonribosomal peptide synthase tyrosine-decahydrofluorene hybrid containing an interesting 13-membered *p*-cyclophane ring. Isolated from the plant-derived fungus *Xenocremonium sinensis*, it inhibits NO production in LPS-induced RAW264.7 cells (IC50 45.8 ± 0.5 µM) [38]. Furanasperterpene A (**5**), a metabolite isolated from *Aspergillus terreus* GZU-31-1, targets the *β*-catenin signaling pathway, exhibiting an anti-adipogenic effect and potential as a lead compound for anti-obesity drugs [39]. Ochroline A (**6**), produced by *Bionectria ochroleuca* SLJB-2, an endophytic fungus from the plant *Selaginella doederleinii*, inhibits the growth of A549, HeLa, and MCF-7 cells. This rare-caged metabolite, featuring a seven-membered ring, is pointed as a structural factor capable of increasing cytotoxicity compared to other indole diketopiperazines with 6/5/6 skeleton [40].

Molecules with new skeletons or unusual functional groups are expected to present differentiated electronic and steric characteristics, enabling unique interactions with biological receptors. These novel molecular recognitions can lead to new mechanisms of action. Consequently, the induction or regulation of new metabolite production is of greatest interest to meet the pharmaceutical industry’s demand for innovation, which faces issues related to cost, efficacy, toxicity, and pharmacokinetics of existing drugs [41]. To address this need, novel approaches have been employed to increase, modify, and diversify secondary fungal metabolism, contributing to expanding the scope of pharmacological activity, and improving the cost–benefit ratio. The OSMAC (one strain, many compounds) strategy refers to random approaches that explore a microorganism´s ability to produce different metabolites in response to changes in the fermentation process [42], including alterations in growing conditions [43], and the addition of biotic and abiotic challenges to the culture medium [44,45]. Jia et al. emphasize the impact of carbon and nitrogen sources, temperature, pH, metal ions, oxidative stress, and fermentation time on the production of secondary metabolites. They highlight the importance of understanding the regulatory mechanisms of biosynthesis to propose strategies that enhance metabolite production for effective application [46].

**Figure 2:**
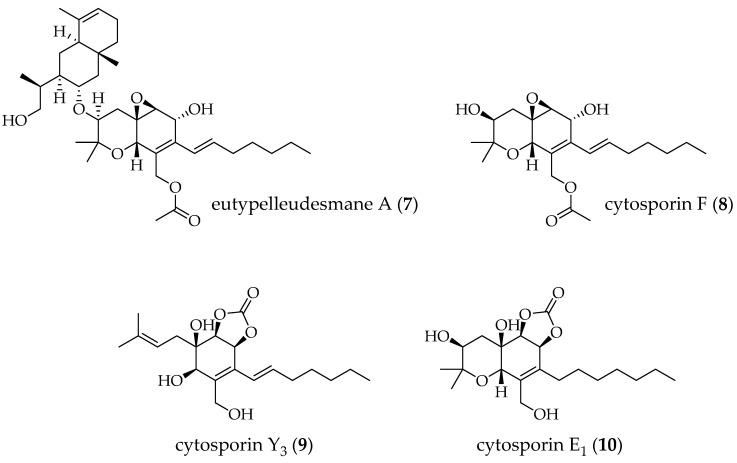
Structures of eutypelleudesmane A, cytosporin F, cytosporin Y3, and cytosporin E1.

However, the production of specific classes of bioactive secondary metabolites by fungi cannot be merely correlated with the fermentative conditions without considering the biosynthetic routes present in each fungal species. This correlation is widely demonstrated, as in the work of Staropoli et al. (2023) [47], which reported that light, shaking, and culture medium promoted the biosynthesis of a chromone by *Trichoderma harzianum*, while increasing the complexity of the culture medium led to the upregulation and downregulation of other metabolite biosynthesis. Achimón et al. (2021) [43] demonstrated the correlation of different carbon sources (glucose, fructose, xylose, sucrose, and lactose) in the production of naphtoquinone pigments by Fusarium verticillioides in submerged cultures. Some metabolites such as eutypelleudesmane A (**7**) and cytosporin F (**8**) were produced only when *F. verticillioides* was grown in rice medium, whereas cytosporin Y3 (**9**) and E1 (**10**), containing rare cyclic carbonate moieties were detected only in a chemically defined culture medium supplemented with l-ornithine hydrochloride [48] (Figure 2). In this context, the production of new fungal metabolites has been the target of research of several approaches, such as the addition of epigenetic modulators during fungal growth.

## 3. Epigenetic-Induced Production of Secondary Metabolites by Fungi

It is estimated that only 25% of secondary metabolites are expressed in vitro [49] due to the constriction of transcriptional regulation, which decreases metabolite production by fungi [50]. In recent decades, significant efforts have been directed towards targeting chromatin, as this complex of DNA and histone proteins influences DNA accessibility, thereby altering the expression of some biosynthetic gene clusters [29]. Covalent modifications of core nucleosomal histones, such as acetylation, methylation, phosphorylation, glycosylation, and rearrangements, can regulate gene transcription, by promoting alterations in the DNA status [51]. Thus, epigenetic-induced regulation of gene expression is a valuable tool for discovering new fungal metabolites. Xue et al. (2023) reported that the use of chemical epigenetic modifiers was responsible for the production of about 540 secondary metabolites in the period of 2007–2022 and presented 25 small organic molecules capable of altering fungal biosynthesis [52].

A deep understanding of chromatin-based modifications in biosynthetic gene clusters can guide the development of fast and effective biotechnological methodologies for producing novel fungal secondary metabolites [53]. Research in this area has focused on two fundamental aspects: optimizing the production of metabolites of interest and activating silent biosynthetic pathways. Optimization is an important approach since secondary metabolites are generally produced at low yields. Sun et al. (2024) [1] reported the isolation and identification of 28 complex secondary metabolites from *Aspergillus* sp. SY2601, with yields between 0.4 and 11.5 mg, while Chen et al. (2024) [35] isolated and identified 34 metabolites (1.0–6.8 mg) from *N. terricola* HDF-Br-2. Epigenetic modifiers can either increase or decrease the yield of secondary metabolites, including mycotoxins, depending on the type and concentration of the modulator used [52]. For instance, Chen et al. (2019) reported a quantitative increase in ergot alkaloids produced by *Claviceps purpurea* with the increased concentration of suberoylaniline hydroxamic acid (SAHA) in the fermentation medium [54]. Even though modern nuclear magnetic resonance (NMR) spectrometers can analyze small quantities of natural products isolated from crude extracts, further exploration of their pharmacological potential is often hindered by low yields. Fungal metabolites currently produced by biotechnological routes for industrial use are the result of yield increases achieved through conventional changes in cultivation parameters, manipulation of specific biosynthetic routes, and microbial engineering [31].

## 4. Protocols and Methodologies for Experiments with Small Molecules Elicitors

Pathway-specific regulators that activate silent gene clusters for the production of novel fungal secondary metabolites are systematically applied as an effective avenue for discovering new drug leads. However, it must be observed that the experimental protocols reported in the literature for biosynthesis activation are highly diverse. The protocols reported to induce or repress the biosynthesis of secondary metabolites by fungi appear to be more related to the experience and standardized procedures of individual research groups than related to the specific general experimental protocols for the activation of new secondary metabolite biosynthesis.

Fungi of the genus *Aspergillus* are used in this review as a good example of species with silent biosynthetic gene clusters involved in the biosynthesis of secondary metabolites. Genome sequencing has demonstrated that the biosynthetic capability of species from this genus exceeds the number and structural diversity of secondary metabolites already reported from chemical prospecting [55]. A recent review by Jia et al. (2024) [46] on strategies to promote gene cluster regulation for the enhancement of secondary metabolite production in *A. oryzae* detailed the molecular regulatory mechanisms for biosynthesis activation, covering effects of transcriptional, epigenetic, and environmental signal regulation. The sequencing of the *A. oryzae* genome enabled remarkable advances in identifying clusters responsible for the biosynthesis of bioactive metabolites of medicinal and industrial interest. The authors provided a comprehensive review of pathway-specific transcription factors and global regulatory factors in *A. oryzae* [46]. *A. awamori*, *A. bombycis*, *A. calidoustus*, *A. fumigatus*, *A. terreus*, *A. versicolor*, *A. westerdijkiae* are some species of the *Aspergillus* genus that responded positively to the stimuli of epigenetic modifiers, activating and regulating biosynthetic routes of the silent epigenome [56].

Table 1 presents the fermentation conditions, as well as the types and concentrations of different epigenetic modifiers, used to modulate the biosynthesis of secondary metabolites in some fungi from the *Aspergillus* genus. Some experiments reported the use of two or more modulators simultaneously. The compound 5-Azacytidine (5-AZA) is frequently used to modulate the secondary metabolism of *Aspergillus* species due to its capacity to promote the reactivation of cryptic fungal biosynthetic gene clusters (BGCs) [57]. However, the parameters related to the use of 5-AZA in the production of secondary metabolites vary greatly. In experiments with *A. clavatus*, the final concentration of 5-AZA was 5 μM [58], but a concentration ten times higher (50 μM) was used for *A. terreus* [59], and even 20× higher (100 μM) in experiments with *A. sydowii* [60]. Mixtures of 5-AZA and suberohydroxamic acid (SBHA) were used with *A. fumigatus* [61] and *Aspergillus* sp. [62]. However, the concentration used in the experiment with *A. fumigatus* was lower than that used for *Aspergillus* sp. (1 mM each) showing lack of standardization even when using the same pair of modulators.

SAHA is another example of a modulator used in dissimilar fermentation parameters. Low concentrations were explored in experiments with *A. niger* (10 μM) [63], *A. versicolor* (10 μM) [64], and *A. wentii* (20 μM) [65], but much higher concentrations were used in experiments with *A. nidulans* (100 μM) [66]. Very different concentrations were used in the biosynthesis activation of *A. terreus* (100 μM [67] and 500 μM [68]). The concentration of 500 μM was also used for metabolite elicitation in *Aspergillus* sp. [62].

**Table 1:** Fungal metabolites isolated from different species of the *Aspergillus* genus under epigenetic modulation conditions.

Fungi Species [Conditions]	Modulator * (Concentration)	Metabolites Detected/Isolated (Bold Numbers Refer to Figure 3)	Ref.
*A. clavatus* [25 °C, 150 rpm, 72 h]	5-AZA ^A^ (5 µM); BUT ^B^ (5 µM); TCA ^B^ (5 µM)	cytochalasin E (**11**) (↑) pseurotin A (**12**) (↑)	[58]
	AGM^A^ + 5-AZA (5 µM each)	pseurotin A (**12**) (↑)	
	VPA ^B^ (5 µM)	cytochalasin E (**11**) (↑) pseurotin A (**12**) (↑)	
*A. fumigatus* [28 °C, static, 30 days]	5-AZA + SBHA ^B^ (500 µM each)	helvolinic acid (**13**) (↨) koaburaside (**14**) (↨)	[61]
*A. fumigatus* GA-L7 [28 °C, 100 rpm, 8 days]	VPA (500 µM)	fumiquinazoline C (**15**) (↑)	[69]
*A. niger* ATCC1015 [25 °C, static, 2 weeks]	SAHA (10 µM)	nygerone A (**16**) (↑)	[63]
*A. nidulans* [37 °C, 250 rpm, 96 h]	SAHA (100 µM)	sterigmatocystin (**17**) (↓) fellutamide B (**18**) (↑) antibiotic 1656 G (**19**) (↑) antibiotic 3127 (**20**) (↑)	[66]
*Aspergillus* sp. SCSIOW2 [28 °C, static, 15 days]	5-AZA + SBHA (1 mM each)	dihydrobipolaroxin D (**21**) ^†^ dihydrobipolaroxin B (**22**) ^†^	[70]
*Aspergillus* sp. AST0006 [28 °C, 160 rpm, 8 days]	SAHA (250–500 µM)	aspyranochromenone A (**23**) ^†^ aspyranochromenone B (**24**) ^†^	[62]
*A. sydowii* [25 °C, 150 rpm, 10 days]	5-AZA (100 µM)	(7*S*)-(+)-7-*O*-methylsydonol (**25**) ^†^ (7*S*,11*S*)-(+)-12-hydroxysydonic acid (**26**) ^†^ 7-deoxy-7,14-didehydrosydonol (**27**) ^†^	[60]
*A. terreus* RA2905 [28 °C, 150 rpm, 7 days]	SAHA (100 µM)	(+) and (−)-asperfuranone (**28**, **29**) asperpyranone A (**30**) asperpyranone B (**31**)	[67]
*A. terreus* GZU-31-1 [rt, static, 30 days]	5-AZA (50 µM)	asperbutyrolactone A (**32**) ^†^ asperbutyrolactone B (**33**) ^†^	[59]
*A. terreus* PF26 [28 °C, 180 rpm, 24 days]	SAHA (500 µM)	(+)-terrein (**34**) (↑) (3*R*)-6-hydroxymellein (**35**) (↑)	[68]
*A. terreus* OUCMDZ-2739 [25 °C, static, 30 days]	TSA ^B^ (10 µM)	(*R*)-4-((2,2-dimethylchroman-6-yl)methyl)-3-(4-hydroxyphenyl)-5-methoxyfuran-2(5*H*)-one (**36**) 1-(2,2-dimethylchroman-6-yl)-3-(4-hydroxyphenyl)propan-2-one (**37**) methyl (*R*)-2-(2-(2-hydroxypropan-2-yl)-2,3-dihydrobenzofuran-5-yl) acetate (**38**) (*R*,E)-3-(2,2-dimethylchroman-6-yl)-4-hydroxy-5-((2-(2-hydroxypropan-2-yl)-2,3-dihydrobenzofuran-5-yl)methylene)furan-2(5*H*)-one (**39**)	[71]
*A. unguis* DLEP2008001 [28 °C, 150 rpm, 20 days]	PRO ^A^ (1 µM)	aspergillusidone F (**40**) (↨) unguinol (**41**) (↨) unguisin A (**42**) (↨)	[72]
*A. versicolor* MCCC 3A00080 [25 °C, 150 rpm, 15 days]	SAHA (20 mg/L)	versiperol A (**43**) ^†^ 2,4-dimethoxyphenol (**44**) diorcinol (**45**)	[73]
*A. versicolor* OUCMDZ-2738 [25 °C, static, 30 days]	SAHA (10 µM)	3-[6-(2-methylpropyl)-2-oxo-1*H*-pyrazin-3-yl]propanamide (**46**) ^†^ (+)- and (−)-brevianamide X (**47**, **48**) (±)-brevianamide R (**49**) (±)-brevianamide Q (**50**) diorcinol C (**51**) diorcinol E (**52**) diorcinol (**45**) methyl diorcinol-4-carboxylate (**53**)	[64]
*A. versicolor* XS-20090066 [rt, 30 days]	SAHA + 5-AZA (100 µM each)	kipukasin K ^†^ (**54**) (↨) kipukasin L ^†^ (**55**) (↨) aspergillusene E ^†^ (**56**) (↨)	[74]
*A. wentii* na-3 [25 °C, static, 30 days]	SAHA (20 µM)	aspewentin A ^†^ (**57**) aspewentin B ^†^ (**58**) aspewentin C (**59**)	[65]

^†^ new compound; (↑) enhanced; (↓) inhibited; (↨) induced; rt: room temperature; * 5-AZA: 5-Azacytidine; AGM: *N*-acetyl-D-glucosamine; BUT: sodium butyrate; PRO: procaine; SAHA: suberoylanilide hydroxamic acid; SBHA: suberohydroxamic acid; TCA = TSA: trichostatin A; VPA: valproic acid. Mechanism of action of the cited chromatin modifiers [52]: ^A^ Inhibition of DNA methyltransferase; ^B^ Inhibition of histone deacetylases of classes I and II.

**Figure 3:**
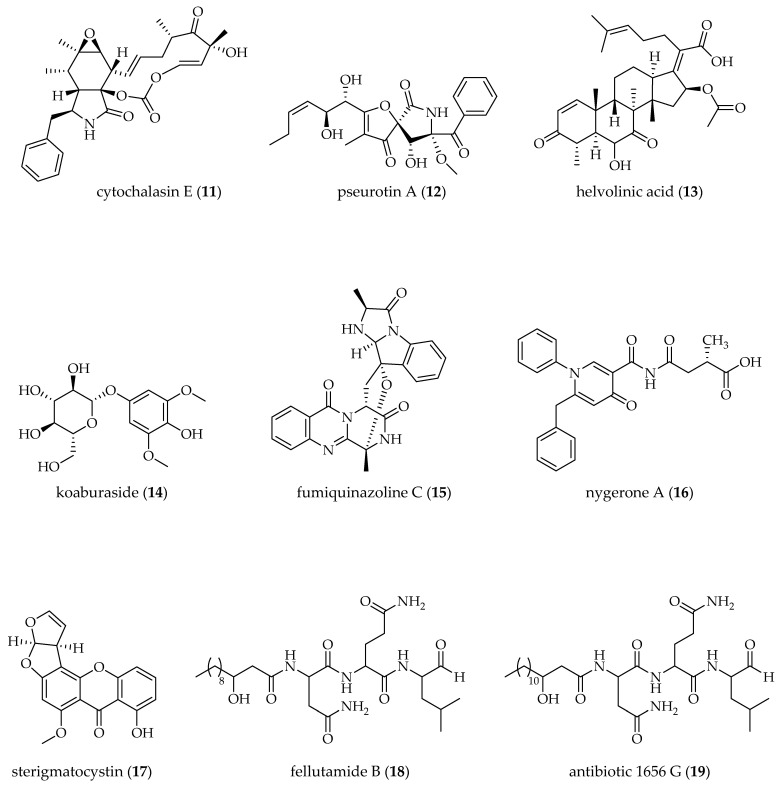

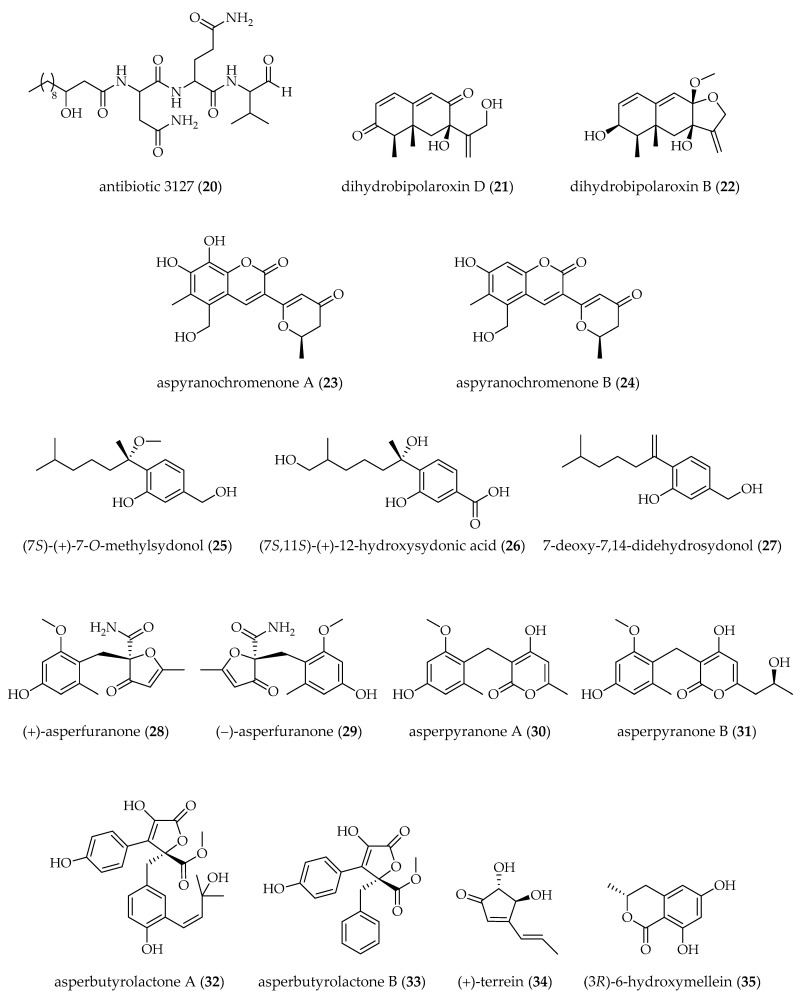

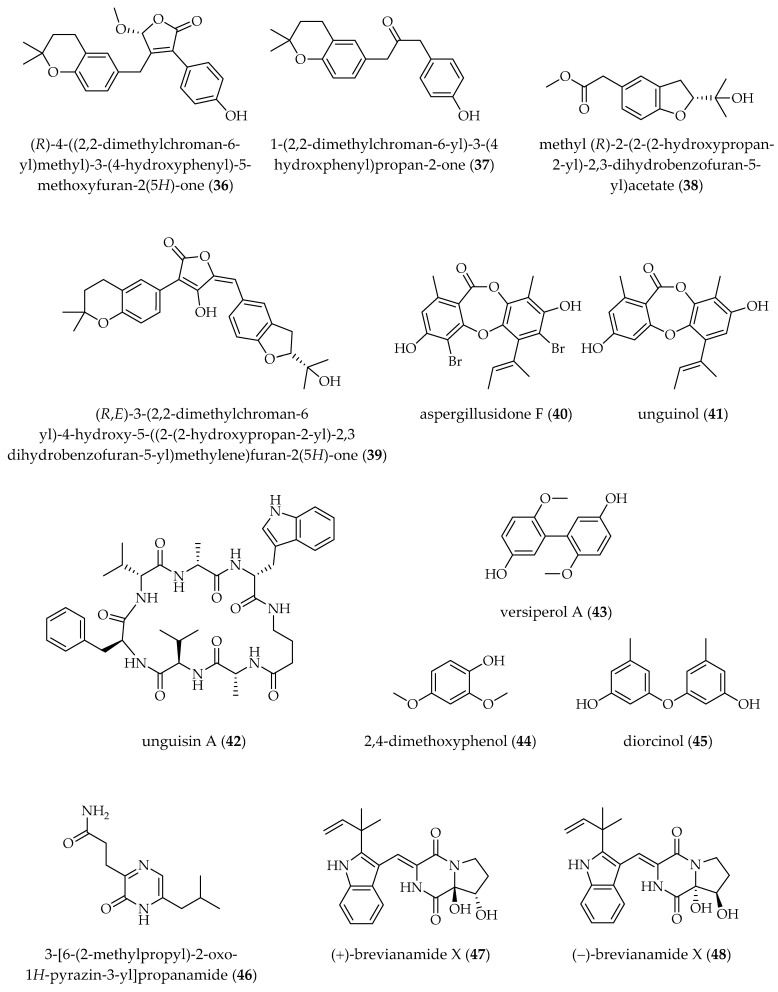

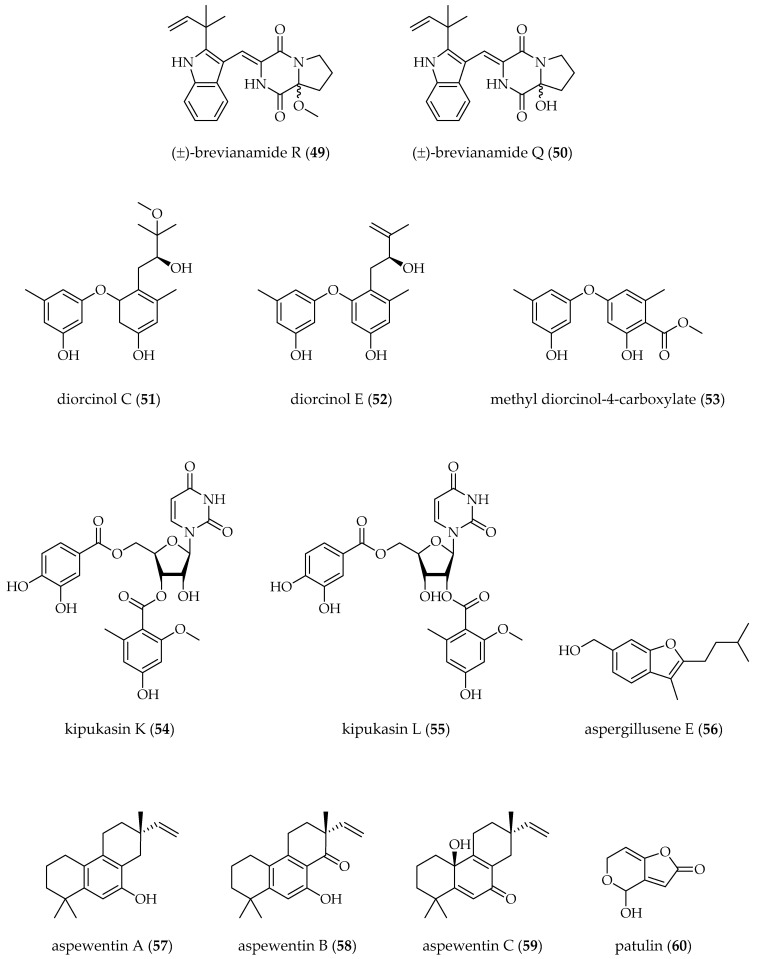
Chemical structures of the metabolites cited in Table 1.

The production of several compounds was described for the species listed in Table 1, exemplifying the diverse possibilities of alteration in the fermentation derived from the action of metabolic modulators (Figure 3). These alterations include increased biosynthesis of one [63,69] or more metabolites already produced by the fungal species [58], production of metabolites not produced in the control [61,72], or biosynthesis of novel metabolites [62,70]. For example, the presence of SAHA during the growth of *A. nidulans* increased the production of fellutamide B (**18**) and antibiotics (**19** and **20**), but restricted production of sterigmatocystin (**17**), indicating different control in the biosynthesis routes [66]. Metabolic changes can be nonspecific, such as the production of pseurotin A (**12**) by *A. clavatus* elicited by different modulators, or selective response, as demonstrated by the biosynthesis of cytochalasin E (**11**), which was not triggered by the mixture of *N*-acetyl-D-glucosamine (AGM) and 5-AZA [58]. Induction of helvolinic acid (**13**) and koaburaside (**14**) production by *A. fumigatus* in response to 5-AZA and SBHA occurs late in the fermentation, while fumiquinazoline (**15**) was readily produced by the same fungal species after 8 days of fermentation [61,69]. *Aspergillus* sp. SCSIOW2 and *A. sydowii* responded to the addition of modulators by producing new sesquiterpenoids of eremophilane-type, dihydrobipolaroxins B (**22**) and D (**21**) [70], and bisabolane-type (**25**–**27**) [60].

In some cases, the response to the epigenetic stimulus has been prolific. For example, in addition to the new metabolites kipukasins K (**54**) and L (**55**), *A. versicolor* produced ten metabolites and significantly enhanced the yield of four nucleoside derivatives in response to the presence of a mixture of SAHA and 5-AZA (100 μM each) in the fermentation [74]. Under the effect of 5-AZA, *A. terreus* produced two new metabolites, asperbutyrolactones A (**32**) and B (**33**), in addition to three known butanolide and four known diphenylether derivatives [59]. The specificity of fungal biosynthesis in response to the stimulus caused by the modulators can be emphasized, for example, in the experiments with *A. terreus*, cultivated by Zhou et al. (2022) [59] and Sun et al. (2018) [71] under very similar conditions (room temperature, static, 30 days). In this case, the use of different modulators, 5-AZA and trichostatin A (TSA), respectively, led to the production of distinct metabolites. Additionally, the metabolic production of *A. terreus*, under the action of the same modulator, SAHA, at different concentrations (100 and 500 μM) and cultivation periods (7 and 24 days), led to different metabolic profiles, producing asperpyranones A (**30**) and B (**31**) [67], terrein (**34**), and hydroxymellein (**35**) [68]. Figure 3 presents the chemical structures of the metabolites cited in Table 1.

In almost all the studies reported in Table 1, the effect arising from the use of the modulators was the increase or activation of metabolic production. However, the results cannot be compared a priori due to the use of such diversified concentrations. This large difference is also observed with other modulators such as TSA (0.5 [58] and 10 mM [71]) and valproic acid (VPA, 5 [58] and 500 mM [69]) in experiments using *Aspergillus* species. The same variation was reported by Xue et al. (2023) [52] reviewing the effect of the DNA methyltransferase modifier 5-AZA on the biosynthesis of 106 secondary metabolites by fungi. The concentration of 5-AZA used by the authors of the 31 papers cited in that review varied greatly, between 1 and 500 μM. Some studies used a concentration of 5-AZA that reached 10 mM, and others worked with concentrations in mg/L (between 10 and 120), showing that, although the experiments were successful, the concentration of the same modulator varied greatly. In the same review, from the 37 works that reported the use of SAHA as a modulator, 140 secondary metabolites were isolated or identified. Again, the concentration of SAHA used in the experiments varied greatly (10–800 μM), 1 mM and 20–80 mg/L [52].

In addition to the concentrations of modulators, most protocols reported in Table 1 use different culture media, varied fermentation times, and both shaking (100–250 rpm) and static experiments. This increases the number of possible protocols, as fermentation time and carbon and nitrogen availability significantly impact the enhancement of metabolite production. Zutz et al. (2013) [58] conducted successful experiments using VPA, TSA, sodium butyrate, 5-AZA, and *N*-acetyl-D-glucosamine to enhance the production of patulin (**60**), cytochalasin E (**11**), and pseurotin A (**12**) by *A. clavatus*. The authors also evaluated the effect of two culture media, which differed in the amount of total nitrogen available to the fungus (0.18 and 0.25 g/L), as well as two fermentation lengths, 48 and 72 h. The literature also reports successful results achieved in short fermentation periods, such as 96 h (*A. nidulans*) [66], while other experiments were kept for 30 days, as reported for *A. fumigatus* [69] and *A. terreus* [67]. The biosynthesis of secondary metabolites in batch cultures can, under certain conditions, start in the trophophase, but typically reaches its peak in the late idiophase and may be modulated by the availability of carbon, nitrogen, phosphate, and other components during the fermentation process. If the amount of carbon is not a limiting parameter, the biosynthesis of secondary metabolites will be maintained until the culture declines [17]. From the viewpoint of prospecting secondary metabolites for technological development and possible industrial use, faster fermentative processes are highly desirable to increase the economic viability of production. Optimizing long fermentation times can be accomplished by following parameters such as depletion of free glucose/carbon source [17]. However, long periods, such as 30 days, have proven to generate novel fungal metabolites even without adding epigenetic modulators [35,40].

## 5. Methodological and Analytical Approaches

The structural diversity and yield of secondary metabolites produced by a fungal species can be assessed without isolating, identifying, and quantifying the metabolites produced. The metabolome can be analyzed using spectroscopic techniques from small amounts of fermented aqueous broths, agar, or organic extracts. Direct analysis of secondary metabolites in the fermented broth is not usual due to the presence of primary metabolites and the matrix effect–catabolism products present in the broth and unutilized nutrients from the culture medium, such as sugars and amino acids, which can hinder the analysis [75]. On the other hand, this methodology has the advantage of not requiring the use of organic solvents since organic extracts are not prepared. Additionally, secondary metabolites are usually produced at very low yields before fermentation parameters are optimized, producing another challenge when analyzing the broth directly. Utilizing microtiter plates can significantly impact the speed of the process, although this procedure requires special care to avoid contamination [76].

The development of methodological conditions for analyzing fermented broth by high performance liquid chromatography (HPLC), for example, can become time-consuming. This is because macromolecules and other components of the nutrient environment can be polar, while the targeted bioactive secondary metabolites are usually small organic molecules with medium polarity [44]. Due to the presence of a diversity of molecules in the broth, comparing it with the unfermented broth profile (as a control) yields variable outcomes since catabolism products cannot be observed in the unfermented condition. Mass spectrometry is one of the most appropriate hyphenated technologies for identifying metabolites present in fermented broths [1]. This technique is advantageous because it requires only a small amount of sample and does not necessitate the preparation of extracts, in addition to being fast.

Smeedsgaard proposed, in the late 1990s, a direct extraction procedure for the rapid standardized analysis of fungal metabolites using a small agar plug where the fungus developed. This methodology, which presupposes the use of a very small volume of solvent, was employed with great efficiency for the HPLC analysis with diode array detector (DAD) of 395 fungal isolates [77]. According to Smeedsgaard’s methodology, 200 mL of extracting solvent was sufficient to prepare the extract. In contrast, in conventional processes, the same volume of solvent would be insufficient for a single extraction of 1 L of fermented broth, as usually three extractions are performed, using an average of 900 mL of extracting solvent per liter of broth [44,77]. Currently, most prospecting experiments report the use of the greener solvent, ethyl acetate (EtOAc), for the extraction of metabolites in the post-fermentation phase [74,78,79]. The use of chlorinated solvents, such as chloroform and dichlorometane, still practiced in various areas such as preclinical medicinal chemistry, is not preferred due to environmental problems and health issues related to these solvents [80]. On the other hand, EtOAc, a green solvent in terms of integration of life cycle assessment and techno-economic analysis, is efficient for the extraction of lipophilic metabolites in works aimed at identifying or isolating small molecules [81]. Large volumes of solvent are usually used during the broth extraction step, but EtOAc is typically recovered for reuse after extraction. Another advantage is that the cost of this solvent is lower than that of other medium polarity solvents, especially chlorinated ones. Nevertheless, good results have been reported using chloroform as an extractor solvent [43].

High-resolution coupled LC-MS systems became one of the main tools for metabolite screening and structural elucidation over the past decades. As their use became more widespread, the improvement of stationary phase resolution for analytical scales became necessary, with the development of smaller particle sizes (e.g., 1.7–2.0 μm) being one of the main advancements over the past 20 years. Several works already described in this review used 1.7 μm C18-columns such as for *A. terreus* [20,39], *X. sinensis* [38], and *Bhushaniella rubra* [17]. Regarding the improvement of resolution when purifying fractions and metabolites from fungi, many studies cited in this review have reported the use of semipreparative and preparative HPLC. Some successful examples can be found in *Aspergillus* sp. [1], *N. terricola* [35], *C.* [36], *A. terreus* [59,67,71], *A. sydowii* [60], *A. fumigatus* [61], *A. versicolor* [64,73,74], and *A. unguis* [72].

Regarding the mass spectrometers, examples of world-class systems include the quadrupole time-of-flight mass spectrometer (Q-TOF-MS), which is nowadays widely used in different branches of natural products chemistry. Some examples highlighted in the current review demonstrate the amplitude of its application in studying fungal metabolites, such as for *A. terreus* [1,20,39,59], *A. fumigatus* [69], *A. unguis* [72], *A. versicolor* [64,73], *X. sinensis* [38], and *T. harzianum* [47]. MS/MS-based systems like Q-TOF-MS have been applied to build molecular networks for investigating the metabolome of fungi. Ultra-high-performance liquid chromatography hyphenated to a diode array detector and a quadrupole time-of-flight mass spectrometer (UHPLC-DAD-Q-TOF-MS) has been involved in combination with NMR data to build molecular networks and to elucidate various compounds. For example, it has been used to study diketopiperazine heterodimers and aspergillicins from *A. caelatus* [82], quinones from strains of different genera [83], and multiple metabolites from 28 *Aspergillus* section Flavi species [84], among other diverse examples. Ultrahigh-performance liquid chromatography coupled with a diode array detector and high-resolution electrospray ionization mass spectrometry (UHPLC-DAD-HR-ESI-MS) and high-resolution electrospray ionization mass spectrometry (HR-ESI-MS) have also successfully identified a diketopiperazine with an unusual skeleton from *Amesia atrobrunnea* and *Polyphilus sieberi*, respectively [85,86].

Sophisticated hyphenated techniques have been used to identify secondary metabolites of fungi, such as high-performance liquid chromatography-high-resolution mass spectrometry-solid-phase extraction-nuclear magnetic resonance (HPLC-HRMS-SPE-NMR) analysis. Wubshet et al. (2013) [87] pioneered the use of HPLC-HRMS-SPE-NMR for matrices from fungi, yielding the identification of six antioxidant metabolites from *Penicillium namyslowskii*. Recently, different research groups have focused on miniaturizing LC systems to improve portability, analysis speed, and reduce the amount of sample required, leading to microfluidics-based liquid chromatographic techniques. Such techniques usually require nanoliter-scale flow, compatible with nanoelectrospray ionization-MS, resulting in highly sensitive systems [88,89]. Danne-Rasche et al. (2020) [90], for example, used a sensitive nano-liquid chromatography/nano-electrospray-MS/MS method (nLC/NSI-MS/MS) to identify more than 800 different lipids from *Saccharomyces cerevisiae*.

## 6. Conclusions

This review provides an overview of the numerous possibilities for obtaining new fungal metabolites by making small changes to the culture medium. The examples corroborate that using chromatin modifiers during the fermentation process can greatly contribute to the production of new metabolites with complex chemical structures and potentially wider scope of biological activities. Although unifying experimental protocols to standardize nutritional sources and physicochemical parameters is difficult, we suggest that the concentrations of chemical epigenetic modifiers be standardized as much as possible. By using the same concentration of modifiers in fermentations, it will be feasible to compare the effects of each modifier on different fungal species in the near future. Therefore, we propose that the effectiveness of epigenetic modulation as a robust and effective tool for inducing the production of novel metabolites for industrial applications depends on the convergence of the numerous experimental methodologies used to modulate fungal secondary metabolism into systematic, standardized, and reproducible protocols.

## Data Availability

Not applicable.
